# Consumer expectations, drug effects, price and purity of heroin and cocaine purchased at drug consumption rooms

**DOI:** 10.1186/s12954-023-00837-3

**Published:** 2023-08-04

**Authors:** Georges Dahm, Karin Roschel, Claude Marson, Adèle Bourmaud, Jennifer Macedo, Mauro Lupo, Lionel Fauchet, Claudia Allar, Raoul Schaaf, Serge Schneider

**Affiliations:** 1 Service de Toxicologie Analytique – Chimie Pharmaceutique, Laboratoire National de Santé, 1, Rue Louis Rech, 3555 Dudelange, Luxembourg; 2 Contact Esch, 130, Rue de Luxembourg, 4344 Esch-sur-Alzette, Luxembourg; 3 Abrigado, 8, Route de Thionville, 2610 Luxembourg, Luxembourg

**Keywords:** Drug consumption room, Harm reduction, Drug checking, Drug quality, Drug price

## Abstract

**Background:**

Drug consumption rooms offer heroin and cocaine consumers a secure and hygienic environment including medical and social guidance. Despite the support and mentoring, only sparse information is available about how drug quality, drug prices and user expectations match at these locations. The present study reports analysis of these three parameters in two drug consumption rooms in Luxembourg.

**Methods:**

Drug users were invited to participate in the project by handing in a few milligrams of the product they planned to consume for chemical analysis and filling out a short questionnaire about the price and their expectations. After consumption, they were asked to report the experienced effects. Drug quality was accessed using LC-Q-ToF and HPLC–UV, and a statistical analysis was carried out of the questionnaires that were correctly filled out.

**Results:**

A total of 513 drug samples have been analyzed. Most consumers were looking for the relaxing/calming effects of heroin and the stimulating effects of cocaine, but they generally overestimated heroin potency and underestimated cocaine potency. No strong correlation based on Spearman’s *ρ* between drug user estimations, drug prices and drug quality was found.

**Conclusion:**

To the best of our knowledge, this study is the first to combine drug analysis with heroin and cocaine user feedback about expectation, drug prices and drug effects. The analytical results were of great interest for users and the staff working at the drug consumption rooms. They may be a strong supplementary communication tool for health care workers when discussing effects and risks of highly toxic substance consumption.

## Introduction

Drug consumption rooms (DCRs), defined as “professionally supervised healthcare facilities where people can consume illicit drugs in a safe and non-judgmental environment” [1], have been established in Western Europe since the beginning of the 1990s. They are low-threshold facilities providing a clean and secure environment for high-risk drug users including needle exchange programs, social counselling and medical care in case of illness or drug overdose [1]. It has been shown that the DCRs have a positive impact on reducing high-risk injection behavior [2], thus lowering morbidity and mortality among hard drug users [3, 4]. In the Grand-Duchy of Luxembourg, two DCRs coexist, located in the capital Luxembourg-City (“Abrigado”) and second-largest town Esch/Alzette (“Contact”). Both have supervised drug injection and inhalation facilities for heroin and cocaine.

On the Luxembourgish drug market, heroin and cocaine prices seem to be declining since several years. According to the national drug report, heroin cost 48 euro per gram (€/g) on average in 2020 [5]. This is in concordance with the 28–70 €/g interquartile range reported on the European market [6]. The price for cocaine was 76 €/g, and the interquartile range reported on the European market in 2020 was 54–83 €/g.

In contrast to recreational drug users [7], little information is available about the quality of drugs consumed at the DCRs and the drug users’ expectations. As the end-users generally cannot know the potency of the product they have purchased, different technics of pre-injection testing have been described [8] such as smoking, snorting or tasting small amounts prior to injection (“slow shots” or “tester shots”).

In this study, heroin and cocaine users were asked to hand over a small part (between 3 and 10 mg) of their samples for chemical analysis to be performed and to fill out a questionnaire regarding their product and expectations. The goal was to determine the chemical quality of drugs consumed at the two DCRs and to correlate these findings to the estimated quality, the reported effects and the price paid for the product.

## Material and methods

### Drug sampling and questionnaire

In 2022, an average of 141 daily consumptions were recorded in the drug consumption facility Luxembourg-City compared to only 14 in the facility Esch/Alzette.

In both facilities, males are largely predominant and the percentage of drug users aged increased during the last years. In 2022, 1% of users were 18–24, 17% were 25–34 and 82% were above 34 years old. Heroin represents about half of consumptions, followed by cocaine and combination of heroin and cocaine. Self-reporting statistics about both facilities are regrouped in Table 1.

**Table 1:** Statistics on drug consumption and user population in 2022 in both facilities

Number of daily consumptions	155
Monthly consumption passages (with ≥ 1 consumption)*	3800
Number of different users per year	800
Male/female ratio (%)	84/16
Heroin in the consumption processes (%)	53
Cocaine in the consumption processes (%)	28
Cocktails of heroin and cocaine (%)	19

*A consumption passage represents a check-in at the DCR, consumption of one or several drugs followed by a check-out. Clients may return several times a day for a new consumption passage

All persons admitted to the DCRs were eligible to take part in the present study. Participation in the project was voluntary, anonymous, linked to comprehensible explanations given by DCR staff members and was not linked to any payment or other advantage.

Once in the consumption room and before consuming the drugs, consumers were supplied with utensils for preparation of their drug (aluminum cooker and aluminum sheets, saline solution, unused syringes and needles, μ-filters, pipes, ascorbic acid and sodium bicarbonate). In the meantime, the social worker explained the study and asked them if they agree to hand in a small amount of drug for chemical analysis. If they agreed, they were also asked to complete a questionnaire.

Before consumption, participants were asked about the nature of the product purchased (heroin, cocaine or another drug), the price paid for the drug (expressed in €/sample or €/g), the preferred consumption mode (intravenous or inhalation) and the expected effects. After consumption, the experienced effects and the estimated potency of the drug (scale from 0 to 100%) were questioned. Samples were analyzed once a week, and feedback was given to the drug users after a maximum of 10 days after collection.

### Chemicals and materials

Heroin (diacetylmorphine), paracetamol (acetaminophen), caffeine, cocaine, phenacetin, levamisole hydrochloride, all at 1 mg/mL were obtained from either Cerilliant (Diegem, Belgium), LGC Standards (Molsheim, France) or Lipomed (Arlesheim, Switzerland). HPLC water, acetonitrile and methanol for HPLC–UV analyses were purchased from Biosolve (Dieuze, France). For LC-Q-ToF analyses, UPLC water with 0.1% formic acid, UPLC acetonitrile with 0.1% formic acid and UPLC methanol were purchased from Fisher Chemical (Merelbeke, Belgium).

HPLC eluents for heroin quantification by HPLC–UV consisted of aqueous buffer adjusted to pH 2.18 using 20 mM potassium dihydrogen phosphate (eluent A) and methanol. A 9/1 (v/v) mixture of A and methanol was used to dilute samples prior to injection. HPLC eluents for cocaine quantification by HPLC–UV consisted of aqueous buffer adjusted to pH 2.18 using solvent A and acetonitrile. A 92/8 (v/v) mixture of A and acetonitrile was used to dissolve and dilute samples prior to injection.

### Qualitative and quantitative analysis

Qualitative analyses were performed using a G6550A ifunnel Q-ToF LC–MS system (Agilent, Waldbronn, Germany) equipped with a 1290 Infinity HPLC system. The system was operated using Agilent MassHunter Workstation. Operating parameters have been published earlier [9].

Heroin and cocaine dosage were performed using an Ultimate 3000 system or Vanquish Flex system (Thermo Fisher, Belgium) equipped with a Dionex Acclaim RSLC PolarAdvantage II column (100 mm × 2.1 mm × 2.2 µm). About 1–10 mg of each sample was weighted exactly and dissolved in 10 mL of methanol using an ultrasonic bath for 5 min. The solution was diluted per 100 in solvent B (heroin), respectively, solvent C (cocaine), and 10 µL of this solution was injected into the HPLC–UV system. The operating parameters have been published earlier [10].

All results are expressed in mass percentage (%, weight/weight).

### Statistical evaluation

Statistical evaluation was performed using Excel datasheets (Microsoft, Redmond, WA, USA). Calculations were conducted only on samples containing the respective data (indication of price, estimated potency, expected and experienced effects). Correlation was estimated using Spearman correlation coefficient *ρ*.

## Results and discussion

Overall, 134 different consumers (17% of the annual population) took part in this study; among those, 106 (79%) were men; 25 women (19%) and 3 (2%) did not indicate their gender.

### Questionnaires

From January 2020 to December 2022, a total of 513 drug samples and questionnaires were collected from the 134 participants among whom several contributed multiple times. Even if many consumers showed an interest in the study, primary regarding potency and presence of contaminants, many questionnaires were not or only partially filled out. The drug users most often declared addictive stress for incompliance or seemed to suffer the effects of drugs or medicines.

A high response rate (93.6%) was only received for the "supposed nature of the product, i.e., heroin, cocaine, mixture of heroin and cocaine or other. Regarding the other questions (Expected, respectively, experienced effects), even if response rates were medium to low (16.0–53.6%), sufficient data could be collected (96 and 82 answers, respectively). Only 24 (4.7%) questionnaires were completely filled out. A summary of the filling quota of the questionnaires is presented in Table 2.

**Table 2:** Amount of data sets provided through questionnaires

Information		Data points collected	No data provided
Bought as	Heroin	319 (62.2%)	33 (6.4%)
	Cocaine	150 (29.2%)	
	heroin + cocaine	8 (1.6%)	
	other (methadone, synthetic THC)	3 (0.6%)	
Price paid for the drug		275 (53.6%)	238 (46.4%)
Expected effects		96 (18.7%)	417 (81.3%)
Experienced effects		82 (16.0%)	431 (84.0%)
Estimated potency		235 (45.8%)	278 (54.2%)

### Expected and experienced effects of heroin and cocaine

*Heroin samples* Fifty-nine (18.5%) heroin users had filled out the “expected effects” field in the questionnaire. The responses were in accordance with well-known psychotropic effects of heroin, such as euphoria, relaxation and analgesic effects. For simplification, responses were merged in four categories:

1. Self-medication (reported as pain reduction, improvement of social interactions, treatment of epileptic attacks, being healthy),
2. Management of addictive stress (reported as relief of withdrawal symptoms),
3. Intoxication (reported as feeling high, experience a flash, being stoned),
4. Relaxation (reported as feeling well, calm or relaxed, not thinking about anything).

Relaxation (26 responses, 44.1%) and intoxication (15 responses, 25.4%) were the most frequently cited expected effects, followed by self-medication (9 responses, 15.3%) and management of addictive pressure (9 responses, 15.3%).

Even if the numbers of responses were low, a trend toward accordance of expected and experienced effects was observed. Twenty-six out of 40 heroin users (65.0%) responded that they experienced the expected effects. The results are summarized in Table 3.

**Table 3:** Heroin users expected versus experienced effects

Expected effect	Responses	Expected effect present*
Relaxation	26	4 Yes, 2 no
Intoxication	15	4 Yes, 1 no
Self-medication	9	1 Yes, 1 no
Management of addictive pressure	9	4 Yes, 2 no
No answer, other answers	260	13 Positive effects, ** 8 negative effects

*Missing numbers: no response given in the questionnaire

**Positive effects: the user reported the expected effect without outlining the exact expected effect

*Cocaine samples* The number of responses was not high enough to enable statistical evaluation. Only 34 users filled out the “expected effects” field in the questionnaire. By far the most often expected effects were a “trip/flash” (26 responses, 76.5%) and a boost of energy (5 responses, 14.7%).

However, 20 out of 33 cocaine consumers (60.6%) reported an experience in accordance with the expectations and 13 out of 33 (39.4%) reported a negative experience. The results are summarized in Table 4.

**Table 4:** Cocaine users expected versus experienced effects

Expected effect	Responses	Expected effect present*
Flash/trip	26	8 yes, 2 no
Energizing	5	0 yes, 1 no
Relaxation	2	0 yes, 0 no
Management of addictive pressure	1	0 yes, 0 no
No answer, other answers	116	12 positive effects, ** 10 negative effects

*Missing numbers: no response given in the questionnaire

**Positive effects: the user reported the expected effect without outlining the exact expected effect

### Estimated and measured drug potency

Overall, 113 and 74 participants reported an estimation of the drug potency for samples containing enough material for analysis and dosage at the laboratory.

The bias between estimated and measured potency was calculated for each sample using following formula:

$${\text{Bias}}\left( \% \right) = {1}00*\left( {{\text{Estimated}}\;{\text{potency}}{-}{\text{Measured}}\;{\text{potency}}} \right)/{\text{Measured}}\;{\text{potency}}$$

Mean heroin potency samples were overestimated by 83%, and the mean cocaine potency was underestimated by about 40% (Table 5), indicating that most heroin and cocaine users only have very limited knowledge about drug levels in the products they buy and consume.

**Table 5:** Estimated potency versus measured potency of heroin and cocaine samples

	Estimated potency (%)			Measured potency (%)			Bias between measured and estimated potency (%)
	Mean	Median	Range	Mean	Median	Range	Median
Heroin (*n* = 113)	32.0	25.0	0–80	16.6	16.5	1.5–56.3	+ 83.4
Cocaine (*n* = 74)	34.8	26.3	0–90	50.5	50.1	8.0–91.6	− 39.9

Very weak correlation (Spearman’s *ρ*: 0.19, *p* < 0.05) was found regarding estimated and measured diacetylmorphine in heroin samples. In 8 out of the 319 samples (1.6%) purchased as heroin, no heroin was detected but cocaine (4 samples), paracetamol only (1 sample), a caffeine/paracetamol mixture (1 sample) or no psychoactive substance at all (2 samples). In eight cases, a heroin content between 0.0 and 1.0% was estimated, but the mean and median potency of these samples (12.7 and 10.6%, respectively) were in similar range then other samples.

For cocaine samples, no correlation (Spearman’s *ρ*: 0.16, *p* = 0.17) toward better quality and higher estimated purity was observed. Nine consumers reported estimated potency levels ranging from 0.0 to 1.0%. Seven samples contained cocaine, the mean and median results were 41.5% and 47.6%, respectively; the range was 18.4–66.2%. MDMA at 10.8% was detected in one sample, and in one other case, the amount of sample received was too low for carrying out quantification.

**Fig. 1:**
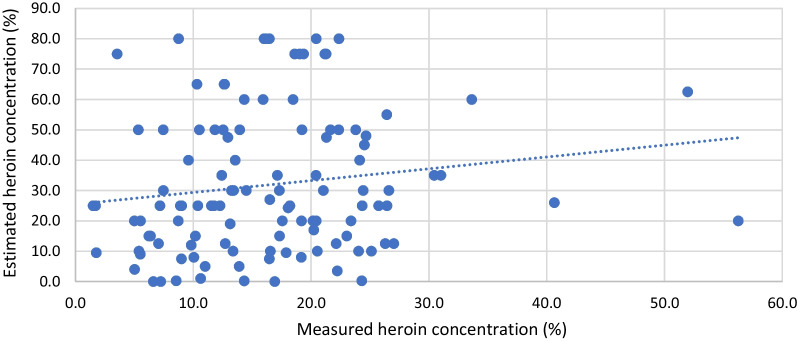
Estimated versus measured heroin potency

**Fig. 2:**
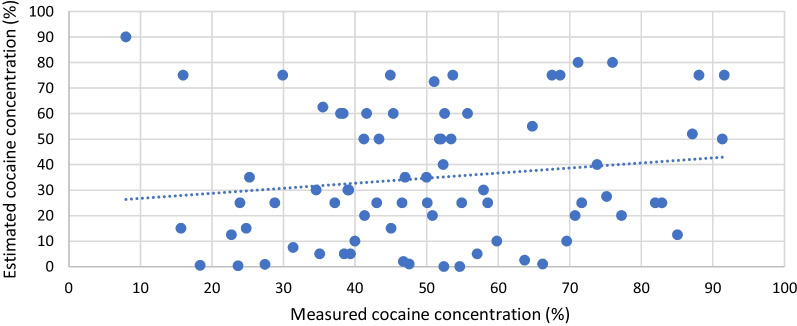
Estimated versus measured cocaine potency

Heroin and cocaine results for estimated versus measured potency are presented in Figs. 1 and 2.

Participants sometimes forgot that they have provided sample in the drug-checking project or where not able to remind the effect of tis specific drug. For this reason, many expressed their desire for faster results, at best before the drug consumption. However, for practical reasons, this demand could not be satisfied. Nevertheless, the results have an influence on the local drug market and on the reputation of the dealers, since drug users explained that they compared the quality of their products.

### Drug price versus drug quality

Overall, 186 (58%) questionaries’ responses indicated a price for heroin and 89 (59%) reported a price for cocaine. Some users indicated the price of the product in €/g and others indicated the price in €/sample making the price/potency evaluation quite difficult. All prices that were given per sample were converted to prices per gram considering a median net weight of 311 mg/sample for both drugs^1^. Some obvious outliers (for example 660 €/g of heroin) have been discarded resulting in 174 heroin and 83 cocaine prices/g used in this study.

The inter-decile price range for cocaine was 48.2–65.5 €/g, the mean price was 56.9 €/g, and the median price was 64.3 €/g. The inter-decile price range for heroin was 21.3–64.3 €/g, the mean price was 44.6 €/g, and the median price was 48.2 €/g.

For heroin, a similar price per gram was found (44.6 €/g) as reported in the national drug report (47.6 €/g) and a lower price was found for cocaine (56.9 €/g) compared to the price given in the national drug report (76.0 €/g, respectively) [5]. The lower cocaine prices at the drug consumption facilities may be the consequence of a socially and geographical distinct market for an underprivileged clientele, compared to the prices paid by other more privileged recreational cocaine users, which is driving up the general average prices of cocaine reported by the Luxemburgish national drug report.

**Fig. 3:**
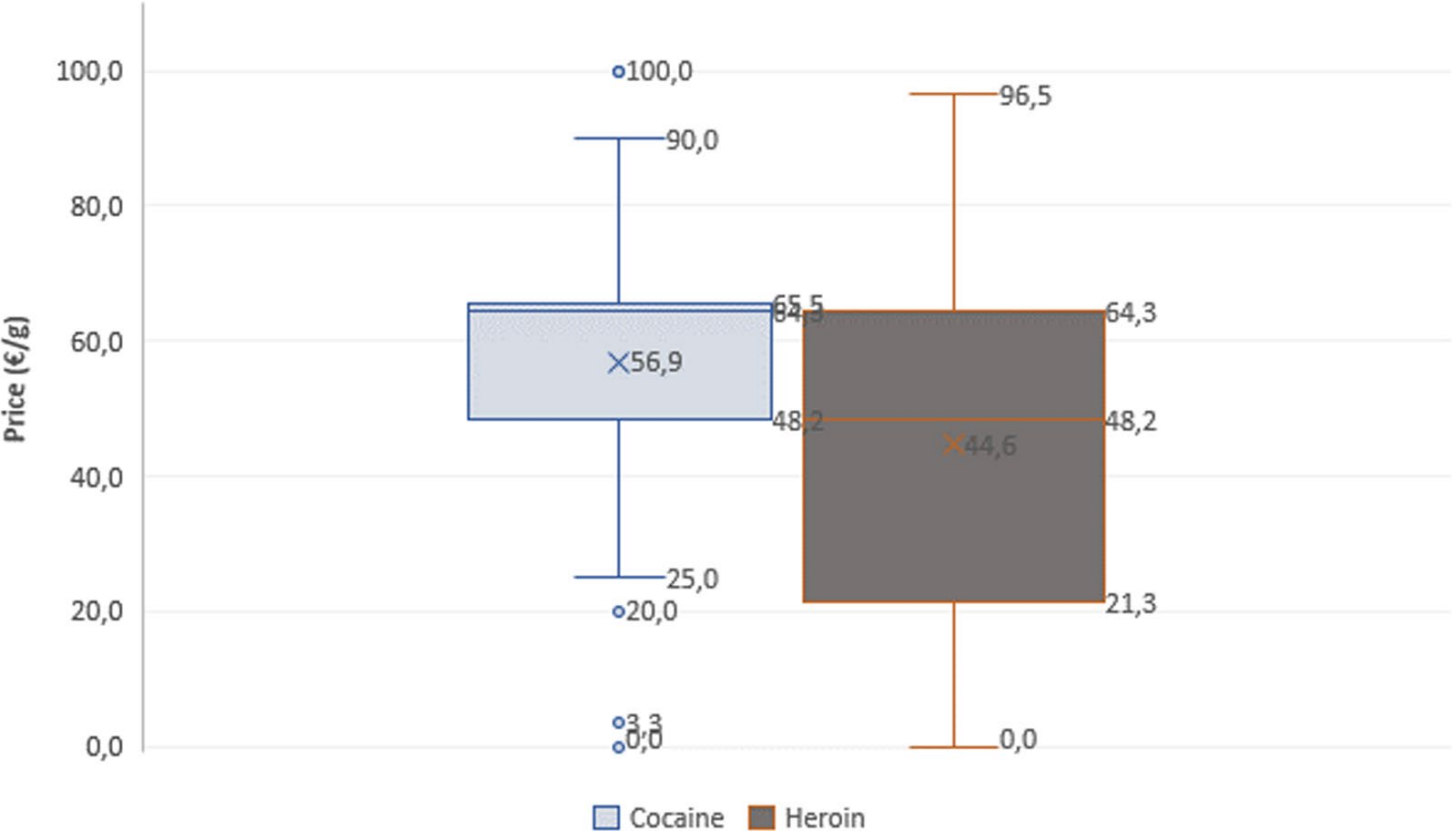
Price (€/g) of cocaine and heroin samples

A summary of the results is presented in Fig. 3.

For heroin, no correlation between price and measured potency (Spearman’s *ρ*: − 0.12, *p* = 0.15) or between price and estimated concentration (Spearman’s *ρ*: − 0.07, *p* = 0.52) was observed. The same conclusion was obtained regarding cocaine (price versus measured potency: Spearman’s *ρ*: 0.22, *p* = 0.07; price versus estimated potency: Spearman’s *ρ*: 0.19, *p* = 0.13).

**Fig. 4:**
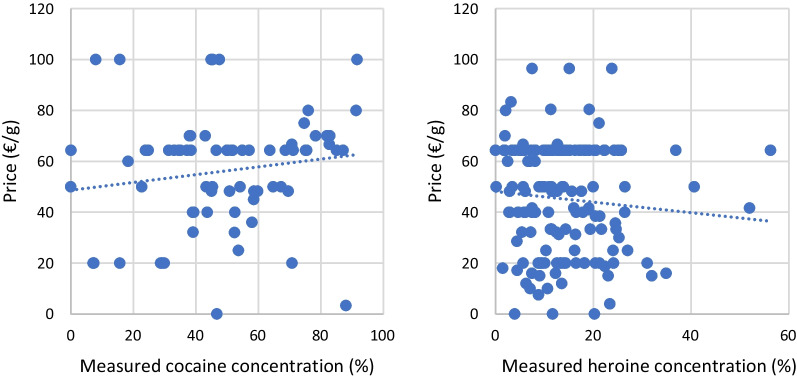
Drug price (€/g) versus drug quality (%)

The price quality relationship for cocaine and heroin is presented in Fig. 4.

## Conclusion

To the best of our knowledge, this is the first study correlating drug user expectations, drug prices and drug quality quantified by a specialized laboratory.

Among drug users, the interest in participating in the study was high, particularly at the beginning of the project. Overall, 17% of total population of individual consumers participated in the study and, if desired, a personal feedback on the drug quality was provided.

When consuming their drug, clients face two challenges regarding the dosage: an unknown potency of the drug but also an estimation of the mass of product. Thus, for the consumers, the interest to participate in the project was to obtain information regarding drug potency, presence of impurities and receiving warnings in case of unusual or dangerous products that may promote overdose or lead to other complications (fentanyl’s, xylazine,…).

For the DCRs, drug checking represents a reliable tool to access market tendencies, price variations and consumer expectations. It offers also a new easy and interesting way to start an open discussion with their clients. Indeed, they can speak about their expectations, fears, feelings, and it offers DCRs employee the possibility to eventually stop rumors about drug quality and adulterants.

The results show that knowledge among heroin and cocaine users regarding the drug potency was low. An 80% relative bias in heroin potency overestimation, and a 40% relative bias in cocaine underestimation has been observed. During discussions, several drug users expressed their astonishment about the relatively high cocaine concentrations, but, on the other side, the inferior quality of the heroin samples did not surprise most consumers.

Expected effects for heroin and cocaine were met by roughly two thirds of responders. No correlation between heroin or cocaine potency and not fulfillment of the expectations was found. Finally, the drug quality did not seem to influence the price or correlate with the drug price. Other factors like drug availability may cause the observed price fluctuations.

The study has some limitations. As only small amounts of sample could be analyzed, inherent inhomogeneity of the sample may give unrepresentative results. No investigation on the motivation of participants to hand in a small amount of their sample was done. It may be an honest interest in drug quality and a way to receive a kind of “quality control” for their drugs. A bias in sample selection may have been introduced if the participants handed over their product only if they had doubts about the quality, as for example a change in drug dealer or a disappointing experience with a previous sample from the same dealer. Finally, many questionnaires were not completely filled out, making a statistical evaluation sometimes difficult. The study will be continued over the next years to extend data collection and refine the findings. Temporal evolutions also will be highlighted with a longer retrospective.

Nevertheless, this study gave a first insight in drug consumers expectations regarding the products they were about to consume, their knowledge about drug potency and the possible relationship between drug potency and drug prices.

## Data Availability

Data will be shared upon request.

## Footnotes

1 Mean net weight was estimated using seized samples from 2019 to 2022. For heroin samples (n = 498, range: 0.13 – 997 mg, mean: 348 mg), a median weight of 311 mg, for cocaine (n = 1344, range: 0.12 – 989 mg, mean: 346 mg) a median weight of 311 mg too was determined in the laboratory.
